# Anti-SARS-CoV-2 Viral Activity of Sweet Potato Trypsin Inhibitor via Downregulation of TMPRSS2 Activity and ACE2 Expression In Vitro and In Vivo

**DOI:** 10.3390/ijms25116067

**Published:** 2024-05-31

**Authors:** Wen-Ping Jiang, Jeng-Shyan Deng, Chia-Chen Yu, Jaung-Geng Lin, Guan-Jhong Huang

**Affiliations:** 1 Department of Pharmacy, Chia Nan University of Pharmacy and Science, Tainan 717, Taiwan; wpjiang@gm.cnu.edu.tw; 2 Department of Food Nutrition and Healthy Biotechnology, Asia University, Taichung 413, Taiwan; dengjs@asia.edu.tw; 3 Department of Chinese Pharmaceutical Sciences and Chinese Medicine Resources, College of Chinese Medicine, China Medical University, Taichung 404, Taiwan; jane224197766@gmail.com; 4 School of Chinese Medicine, College of Chinese Medicine, China Medical University, Taichung 404, Taiwan; jglin@mail.cmu.edu.tw; 5 Chinese Medicine Research Center, China Medical University, Taichung 404, Taiwan

**Keywords:** sweet potato, trypsin inhibitor, SARS-CoV-2, ACE2, TMPRSS2

## Abstract

Severe acute respiratory syndrome coronavirus 2 (SARS-CoV-2) has caused a global pandemic. Known as COVID-19, it has affected billions of people worldwide, claiming millions of lives and posing a continuing threat to humanity. This is considered one of the most extensive pandemics ever recorded in human history, causing significant losses to both life and economies globally. However, the available evidence is currently insufficient to establish the effectiveness and safety of antiviral drugs or vaccines. The entry of the virus into host cells involves binding to angiotensin-converting enzyme 2 (ACE2), a cell surface receptor, via its spike protein. Meanwhile, transmembrane protease serine 2 (TMPRSS2), a host surface protease, cleaves and activates the virus’s S protein, thus promoting viral infection. Plant protease inhibitors play a crucial role in protecting plants against insects and/or microorganisms. The major storage proteins in sweet potato roots include sweet potato trypsin inhibitor (SWTI), which accounts for approximately 60% of the total water-soluble protein and has been found to possess a variety of health-promoting properties, including antioxidant, anti-inflammatory, ACE-inhibitory, and anticancer functions. Our study found that SWTI caused a significant reduction in the expression of the ACE2 and TMPRSS2 proteins, without any adverse effects on cells. Therefore, our findings suggest that the ACE2 and TMPRSS2 axis can be targeted via SWTI to potentially inhibit SARS-CoV-2 infection.

## 1. Introduction

The World Health Organization declared the COVID-19 pandemic a global public health emergency, representing one of the worst epidemics that humanity has faced in decades, with high rates of morbidity and mortality [1]. Countries around the world implemented various measures to mitigate the impacts of the disease, such as reducing interpersonal contact, but their effectiveness was limited due to the virus’s spread via asymptomatic carriers. Although several treatment options were proposed and tested, most required reliable clinical trial data before they could be widely and safely used. Although vaccines and drugs have been developed for COVID-19, the management of critical cases through respiratory therapy, antiviral therapy, and immunotherapy remains highly challenging. However, clinical trial data are necessary for the broad and safe use of most treatments [2].

SARS-CoV-2 utilizes the ACE2 and TMPRSS2 proteins located on the surfaces of host cells to gain entry into human cells. ACE2 acts as a receptor and serves as a possible route by which the coronavirus can enter cells, while TMPRSS2 is a membrane proteinase that can aid the virus in entering cells. The ACE2 protein is the cause of the lung infection triggered by SARS-CoV-2; it is mainly expressed on the surfaces of the human respiratory tract and lung cells [3]. When the virus enters the host cells, TMPRSS2 cuts the virus membrane protein, promoting its replication and infection, and it is mainly located on the surfaces of human respiratory tract cells [4]. ACE2 and TMPRSS2 are critical for the entry of SARS-CoV-2 into host cells and represent important mechanisms of COVID-19 infection. Therefore, understanding these proteins through research could facilitate the development of effective drugs to treat COVID-19 [5]. The ACE2 protein is mainly expressed in lung tissue and can also be detected in other tissues throughout the body, including the heart, kidneys, digestive system, vascular endothelial cells, central nervous system, and skin [6]. The TMPRSS2 protein is mainly expressed in airway epithelial cells but can also be found in other tissues, such as the prostate, pancreas, kidneys, small intestine, heart, and lymphocytes. The differential expression of these two proteins in different tissues may affect SARS-CoV-2 infection [7]. Moreover, SARS-CoV-2 infection can lead to immune disorders, which may be due to the release of a large number of viruses after infecting host cells, activating the host immune system and causing inflammation [8]. Further research is needed to gain a deeper understanding of the pathophysiological mechanisms underlying this phenomenon. An excessive inflammatory response, called a cytokine storm, typically occurs in infectious diseases such as influenza, SARS, and COVID-19 and autoimmune diseases such as rheumatoid arthritis and systemic lupus erythematosus. During viral infection, the virus can stimulate the body’s immune system to produce cytokines to guide the immune response. However, if the immune response is excessive, it can trigger a cytokine storm, resulting in a widespread and severe inflammatory response and tissue damage. A cytokine storm, a severe complication associated with certain diseases, can induce multi-organ dysfunction and, in the most severe cases, cause death, highlighting its grave consequences [9,10].

Protease inhibitors function by inhibiting the activity of proteases, enzymes that are crucial for several biological processes, such as digestion, blood clotting, and immune system regulation. In the case of viruses, proteases are essential for their replication and maturation, including those of COVID-19, HIV, and hepatitis C. Therefore, the use of protease inhibitors as antiviral drugs can prevent or slow down virus replication [10]. Protease inhibitors, including trypsin inhibitors, are typically protein or peptide molecules that are produced by plants, animals, and microorganisms [11]. A trypsin inhibitor is a protein or peptide molecule that can bind to and inhibit the activity of trypsin, an enzyme that plays a role in protein digestion and absorption. The function of the trypsin inhibitor in plants is frequently attributed to its role as a defense protein; it helps to deter predation and parasitism by insects or other animals [12]. SWTI is known to possess numerous health benefits, such as antioxidative, anti-inflammatory, ACE-inhibitory, and anticancer properties [13,14,15,16]. By presenting evidence, we support the notion that SWTI has the potential to inhibit SARS-CoV-2 infection by targeting the ACE2 and TMPRSS2 pathways, suggesting that it could be a promising nutraceutical for the development of COVID-19 therapies.

## 2. Results

### 2.1. A Method for the Separation of Sweet Potato Trypsin Inhibitor (SWTI)

**Figure 1:**
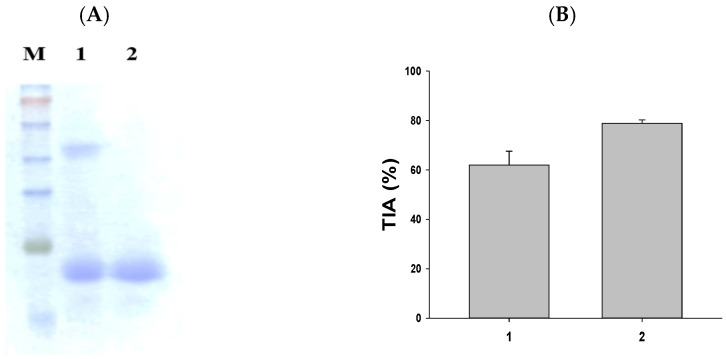
SDS-PAGE of SWTI using aqueous two-phase systems (**A**) and assessment of trypsin inhibitor activity (TIA) (**B**) in sweet potato. Lane 1: crude extract of sweet potato (5 μg). Lane 2: purified SWTI (2 μg).

The purification of SWTI is achieved using aqueous two-phase systems [15]. As illustrated in Figure 1A, the partial purification of SWTI was carried out, followed by protein staining. The resulting image revealed the presence of two protein bands at 50 kDa and 23 kDa (Figure 1A, lane 1). The major trypsin inhibitor band was found to be the 23 kDa band, leading to the use of 10% preparative SDS-PAGE gels for the isolation of the purified SWTI’s 23 kDa TI band (Figure 1A, lane 2) for further experimentation. In addition, the crude extract of sweet potato and purified SWTI exhibited strong inhibitory activity against trypsin in vitro (Figure 1B). As a result, SWTI might fulfill a protective role as a protease inhibitor, while also serving as a storage protein.

### 2.2. The Cell Toxicity Assay Was Conducted Using Different Concentrations of SWTI

**Figure 2:**
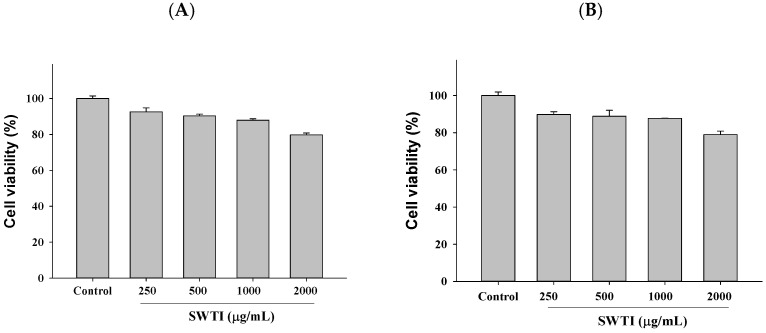
Cytotoxic effects of SWTI on HepG2 (**A**) cells and 293T (**B**) cells. The MTT assay was employed to assess the cell viability after exposure to different concentrations of SWTI (250, 500, 1000, and 2000 μg/mL) for 24 h, to investigate its potential cytotoxicity. Data are means ± S.D. (*n* = 3).

The high expression of the ACE2 and TMPRSS2 proteins was observed in HepG2 (liver cancer) and 293T (human embryonic kidney) cells, which were utilized as screening models to investigate the entry of SARS-CoV-2 into host cells. Subsequently, the inhibitory potential of SWTI (250–1000 μg/mL) against ACE2 and TMPRSS2 in these cells was tested. To ensure the safety of the SWTI in the subsequent experiments, we first evaluated its toxicity to cells using the MTT assay. Based on the results, it was determined that concentrations of SWTI below 1000 μg/mL were non-toxic to cells, leading us to select 500 and 1000 μg/mL for further investigation in this work (Figure 2A,B).

### 2.3. The Expression of ACE2 and TMPRSS2 Was Reduced by Treatment with SWTI

**Figure 3:**
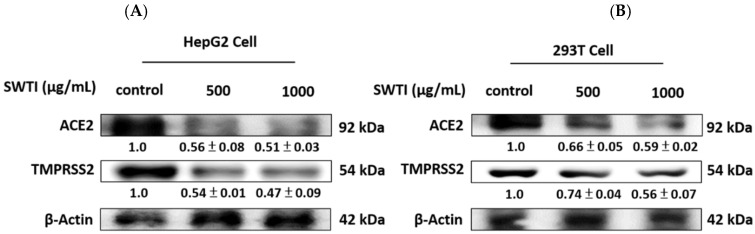
The protein expression of ACE2 and TMPRSS2 was suppressed by SWTI treatment in HepG2 (**A**) and 293T (**B**) cells. After treating the cells with SWTI (500 and 1000 μg/mL) for 24 h, the protein expression levels were analyzed using Western blotting (*n* = 3). Densitometric analysis was used to determine the ratio of protein expression in SWTI-treated cells to that of the control, with β-actin serving as a positive control.

The protein expression of ACE2 and TMPRSS2 is depicted in Figure 3A,B, demonstrating the effect of SWTI. After 24 h of exposure to SWTI, the HepG2, and 293T cells displayed a dose-dependent reduction in the protein expression of ACE2 and TMPRSS2.

### 2.4. The Efficacy of SWTI in Animal Models Was Evaluated

**Figure 4:**
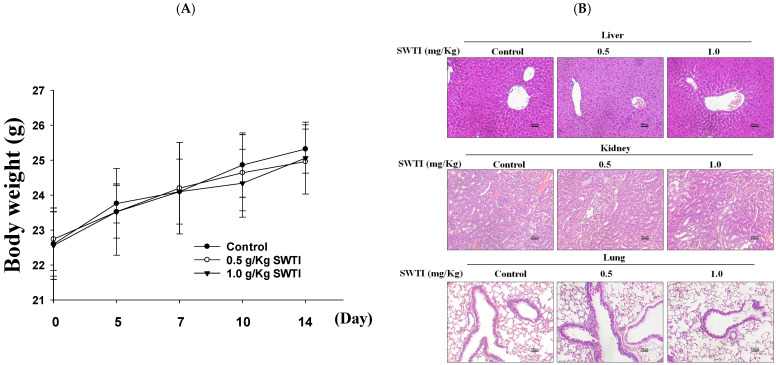
The efficacy of oral SWTI administration was assessed using animal models. (**A**) The mice were pretreated with 0.5 and 1.0 g/kg SWTI, and their body weights were measured, as well as capturing H&E staining images of their livers and kidney tissue (200×; scale bar = 100 μm). (**B**) H&E staining was performed on mouse liver, kidney, and lung tissue sections, and representative histology images were taken at high magnification. Data are means ± S.D. (*n* = 6).

An evaluation of the in vivo effectiveness of oral SWTI was conducted using an animal model. Mice were administered 0.5 and 1.0 g/kg of SWTI orally for 14 days as a pretreatment. The results showed that the administration of oral SWTI for 14 days did not lead to significant changes in the body weights of the mice (Figure 4A) and did not induce toxicity in the liver, kidneys, or lungs, as demonstrated via H&E staining (Figure 4B).

### 2.5. Immunohistochemical Images Were Used to Demonstrate the Inhibitory Effects of SWTI on ACE2 and TMPRSS2 Protein Expression

**Figure 5:**
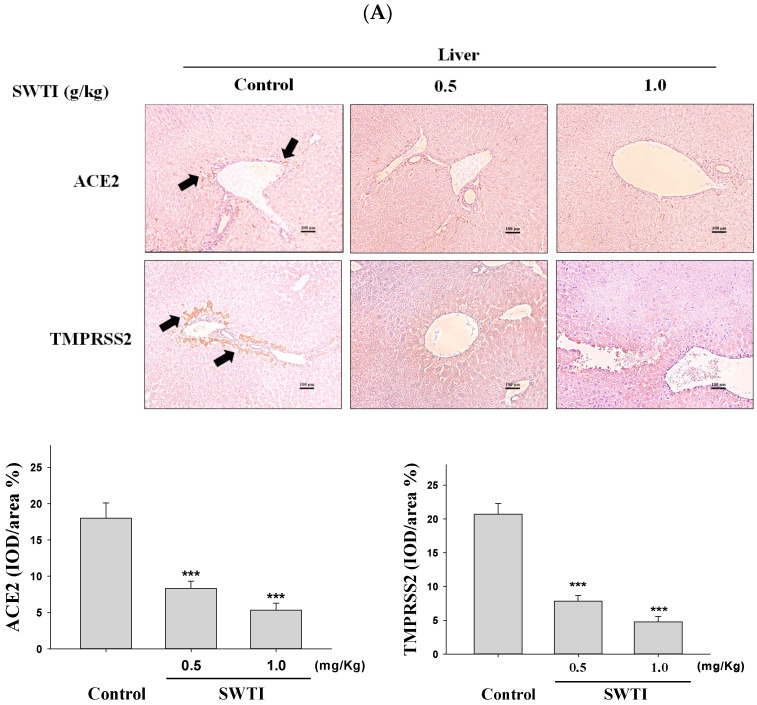

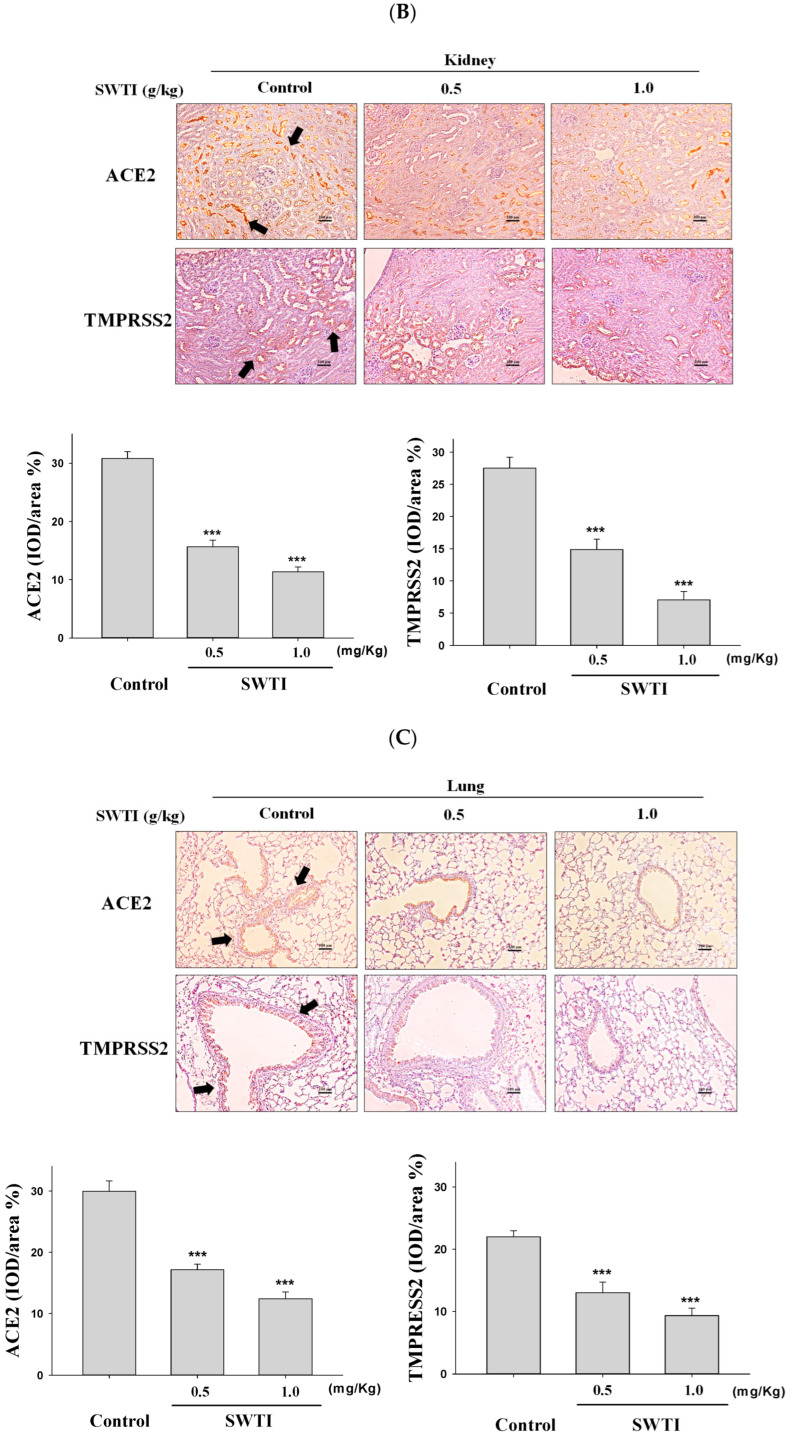
ACE2 and TMPRSS2 expression was inhibited by SWTI in animal models. The effects of oral SWTI administration on ACE2 and TMPRSS2 expression in liver (**A**), kidney (**B**), and lung tissues (**C**) were analyzed using immunohistochemical staining. Mouse liver and kidney sections were magnified and photographed to obtain representative histology images after H&E staining. The data are presented as the IOD/area (%) in the study. Data are the mean ± S.D. (*n* = 6). *** *p* < 0.001 vs. control group. The arrows indicate the expression of ACE2 or TMPRSS2. Scale bar = 100 μm.

The presence of ACE2 and TMPRSS2 expression in tissues was determined via immunohistochemical analysis. The administration of SWTI at doses of 0.5 and 1.0 g/kg resulted in a decrease in the expression of ACE2 and TMPRSS2 in the liver, kidney, and lung tissues (Figure 5A–C). By demonstrating the effective suppression of the expression of the ACE2 and TMPRSS2 proteins in liver, kidneys, and lungs, our results provide compelling evidence for the use of SWTI as a viable candidate to prevent SARS-CoV-2 infection and transmission.

### 2.6. The Western Blot Analysis Demonstrated That SWTI Treatment Effectively Inhibited the Protein Expression of ACE2 and TMPRSS2

**Figure 6:**
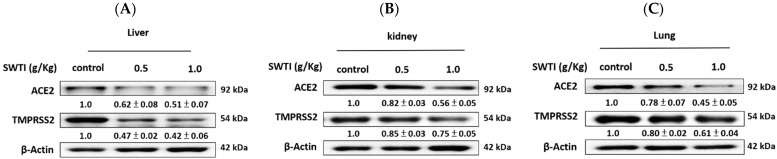
ACE2 and TMPRSS2 protein expression in the liver (**A**), kidney (**B**), and lung (**C**) tissues of mice were inhibited by SWTI administration. Densitometric analysis of the protein bands from the Western blot analysis was used to determine the ratios (SWTI/control) of ACE2 and TMPRSS2 expression in the liver and kidney tissues. Data are the mean ± S.D. (*n* = 3). β-actin was used as a positive control.

The verification of the effects of SWTI on ACE2 and TMPRSS2 protein expression was carried out via Western blot analysis. The levels of ACE2 and TMPRSS2 expression in the liver, kidney, and lung tissues were significantly reduced following pretreatment with SWTI, as demonstrated by the results (Figure 6A–C).

## 3. Discussion

With the global spread of the coronavirus disease (SARS-COV-2), it has become crucial to provide preventive treatments for high-risk groups. The targeting of viral entry through adjuvant therapy has the potential to significantly reduce the disease’s severity and limit viral propagation [17]. The current focus of drug research is on viral proteins, such as remdesivir, or small molecules that target human proteins utilized by viruses, given the urgent need for treatments. The integration of targeted drugs and vaccination strategies may offer a valuable means of addressing particular patient populations or reducing the potential spread of the virus in those who remain unvaccinated [18].

Efforts to identify organs that are more vulnerable to SARS-CoV-2 infection have focused on examining ACE2 protein expression at the protein level, given the wide range of symptoms experienced by individuals affected by the virus. The expression of ACE2 is observed in different organs in the human body, including the lungs, heart, kidneys, and intestines [19]. In particular, ACE2 is abundantly expressed in the lungs and this is one of the major organs in which it is expressed. As the SARS-CoV-2 virus, which causes COVID-19, enters human cells through the ACE2 receptor, the role of this receptor is critical. Due to the high abundance of ACE2 in the lungs, this organ is a prime target for viral infection and can lead to the development of respiratory symptoms [20]. Although the lungs have the highest concentration of ACE2, other organs, such as the heart, kidneys, and intestines, also contain this receptor. This may be one reason that severe cases of COVID-19 can lead to multi-organ damage. The expression and function of ACE2 in different organs are key to providing insights into the pathogenesis of viral infections and COVID-19 [21]. Since most people experiencing SARS-CoV2 infection develop a sore throat, it can be manifested by the release of inflammatory agents, and this inflammation increases the ACE2 expression and the degree of infection in the affected person [22]. Furthermore, among the tissues examined, the bowel displayed the highest ACE2 expression, followed by the kidneys, testis, and gallbladder. Notably, cardiac tissues exhibited the highest level of serum ACE2 expression [23]. Based on the results, it can be inferred that SARS-CoV-2 infection is associated with gastrointestinal disorders and renal dysfunction. AKI is a common complication in patients with SARS-CoV-2 infection, affecting approximately 20% to 30% of cases; it may result from the development of systemic inflammation caused by COVID-19, leading to microvascular disease, glomerular necrosis, and renal tubular epithelial cell damage [24,25]. COVID-19 infection can also cause a decrease in ACE2 expression, leading to the excessive activation of the renin–angiotensin system and cytokine storms, which can result in kidney injury and glomerular microvascular lesions [26]. At present, there are no medications that specifically target ACE2 for treatment. Although ACE2 is the main receptor for the entry of SARS-CoV-2 into host cells, it also plays an important role in maintaining physiological homeostasis. Drugs targeting ACE2 inhibition may therefore have negative effects in humans, including cardiovascular and metabolic disorders [27].

The entry of SARS-CoV-2 into host cells is predominantly mediated by the cleavage and activation of the spike protein by TMPRSS2. This mechanism enhances the fusion between the viral and host cell membranes [28]. The role of TMPRSS2 in enhancing the infectivity of SARS-CoV-2 is of great significance in the pathogenesis of COVID-19 [29]. Camostat mesylate is a serine protease inhibitor that has been found to have various functions, such as the inhibition of tumors, liver fibrosis, and pancreatitis. Recent studies have shown that it can also inhibit the entry of COVID-19 into Caco-2 and Vero195 TMPRSS2 cells. Therefore, TMPRSS2 inhibitors have emerged as a promising therapeutic approach for COVID-19, and numerous studies are currently investigating their efficacy [8].

The ACE2 receptor is located on the cell membrane, present in the lungs, heart, kidneys, and intestinal tissue, and its main function is to degrade angiotensin II and regulate the renin–angiotensin–aldosterone system (RAAS). In addition, by acting as a receptor, ACE2 enables the infiltration of host cells by SARS-CoV-2, causing the onset of COVID-19 viral infection. During infection with SARS-CoV-2, the ACE2 receptor is disrupted, leading to an imbalance in RAAS and angiotensin II [30]. Due to these physiological changes, patients are at an increased risk of serious complications, especially damage to the lungs and heart [31]. Further infection with SARS-CoV-2 may lead to an imbalance and damage in the immune system, which may be one of the reasons for the severe complications that occur in COVID-19 patients [32]. Overall, the interaction between RAAS and ACE2 plays a pivotal role in modulating the host’s susceptibility to SARS-CoV-2 infection. A deeper understanding of the RAAS–ACE2 interaction may offer new therapeutic and preventative strategies for COVID-19.

COVID-19 can cause pathological damage to various organs, such as the lungs, heart, kidneys, and liver. Among these, the lungs are the most commonly and severely affected organs during COVID-19 infection. The damage caused by SARS-CoV-2 infection to the alveolar and bronchial epithelial cells can result in pneumonia, pulmonary edema, and pulmonary fibrosis [33]. Severe pneumonia can lead to acute respiratory distress syndrome (ARDS), a critical illness caused by excessive fluid in the alveoli and damage to the lung tissue, which affects gas exchange and results in hypoxia [34]. The heart is one of the vital organs affected by COVID-19. SARS-CoV-2 infection can cause damage to cardiac cells, increase cardiac stress, and result in myocarditis and myocardial infarction, which may lead to severe complications and even death [35]. The kidneys are also one of the major organs affected by COVID-19. COVID-19 infection can lead to acute kidney injury, with an incidence of 5% to 15%. In severe infections, the host can experience serious complications, such as renal dysfunction and chronic kidney disease [36]. COVID-19 infection can also affect the liver, with approximately 20% to 50% of patients experiencing impaired liver function, including elevated liver enzymes and hepatitis [22]. Overall, SARS-CoV-2 infection exerts a significant impact on multiple organs in the human body. A more comprehensive understanding of the infection mechanism and pathology of SARS-CoV-2 may provide better treatment and prevention strategies.

A comprehensive understanding of SARS-CoV-2’s transmissibility requires a focus on the expression of host cell surface receptors. New variants of SARS-CoV-2 continue to emerge, causing surges, breakthrough infections, and devastating losses—underscoring the importance of identifying SARS-CoV-2 antivirals. A simple, accessible human cell culture model permissive to SARS-CoV-2 variants is critical in identifying and assessing antivirals in a high-throughput manner. Although human alveolar A549 cells are a valuable model for the study of respiratory virus infections, they lack two essential host factors for SARS-CoV-2 infection: ACE2 and TMPRSS2 [37]. The research model utilized in this study includes two cell lines, one of which is the HepG2 cell line. This cell line is known for its non-toxic nature and ability to express various liver-specific proteins, including albumin and cytochrome P450, outside the body [20]. The HEK 293-derived 293T cell line, containing the simian virus 40 large T antigen, is a widely adopted cellular model for protein expression, genetic engineering, and virus production [21]. In an animal model, mice can also be infected with COVID-19. The ACE2 and TMPRSS2 proteins are also expressed in different organs in mice. Hence, in this study, the antiviral activity of SWTI was evaluated using these two cell lines and animal models. The results indicated a decrease in the expression levels of ACE2 and TMPRSS2 in vitro and in vivo.

Endogenous protease inhibitors can be classified into the Kunitz, Kazal, serpin, and mucin families, all of which have the function of inhibiting protease activity. Recent studies have shown that the protease inhibitor system plays a crucial role in the inflammatory process [38]. During inflammation, various cells and tissues release proteases that can cleave and activate other proteins, further triggering the inflammatory response. Excessive protease activity can also lead to tissue damage and inflammation. The protease inhibitor system can regulate the activity of these proteases, thus reducing the inflammatory response and tissue damage [39]. In addition to their role in inhibiting protease activity, Kunitz-type protease inhibitors have also been found to engage in biological activity, such as participating in angiogenesis, nerve development, platelet aggregation, and immune regulation and exerting antiviral, anti-tumor, and antibacterial effects [40]. Therefore, they have broad prospects in drug development. The material studied in this work is SWTI, which belongs to the family of Kunitz-type protease inhibitors, and its antioxidant, anti-inflammatory, ACE-inhibitory, and anticancer properties have been confirmed in various studies [13,14,15].

SARS-CoV-2 relies on the proteases in host cells to process and replicate its proteins. Therefore, the use of protease inhibitors can interfere with the virus’s life cycle in host cells, thereby inhibiting its replication and spread [41]. The proteases in SARS-CoV-2 include the main protease (M^pro^) and papain-like protease (PL^pro^), both of which are necessary for the virus to replicate in host cells. Therefore, the use of protease inhibitors can suppress the activity of these proteases and prevent the virus from replicating and spreading [42]. For example, Remdesivir is a nucleotide analog that is metabolized into its active triphosphate form in the body. It can bind to the RNA polymerase involved in viral RNA replication and inhibit its activity, thereby suppressing the virus’s replication [43]. In addition, Lopinavir and Ritonavir can be used to treat COVID-19 by inhibiting the virus’s main proteases, thereby preventing the virus from replicating and spreading [44]. Overall, protease inhibitors are a treatment method for SARS-CoV-2 that can interfere with the virus’s life cycle in host cells, thereby inhibiting its replication and spread. Although no effective treatment options for COVID-19 have yet been established, SWTIs may be considered as prophylactic agents to prevent the clinical manifestation and disease progression of COVID-19 in susceptible populations.

## 4. Materials and Methods

### 4.1. Purification of SWTI

The storage roots of Tainong 57 sweet potato (*Ipomoea batatas* [L.] Lam cv. Tainong 57) were procured from local wholesalers. The roots were subjected to washing and peeling and were cut into strips before SWTI extraction and purification. The crude extract was added to PEG6000 (11%; *w*/*w*)/phosphate buffer (containing equal molar amounts of K_2_HPO_4_ and NaH_2_PO_4_ at 16.5% each)/KCl (9%; *w*/*w*) at pH 6.0, and then vortexed at 25 °C for 30 min, and the reaction mixture was separated into two phases. It was centrifuged at 5000× *g* for 15 min at 4 °C [13].

### 4.2. Trypsin Inhibitor Activity Assay (TIA)

The substrate used in the TIA assay was N-benzoyl-L-arginine ethyl ester [45]. The samples (50 μg) were preincubated with 4 μg trypsin at room temperature for 15 min, and then the substrate was added and they were further incubated for 20 min. The absorbance at 405 nm was assessed. The TIA was determined through three measurements, and the mean value was expressed as a percentage.

### 4.3. Cell Culture

Human HepG2 cells were provided by the Biological Resources Collection and Research Center (BCRC; Hsinchu, Taiwan) (BCRC number: 60364). The HEK293T cell line was obtained from Hsien-Tsung Yao (Department of Nutrition, China Medical University, Taiwan). Six-well plates were employed to seed 2.5 × 10^4^ cells, and these cells were cultured in a humidified atmosphere with 5% CO_2_ at 37 °C in Dulbecco’s modified Eagle medium (DMEM) supplemented with 10% fetal bovine serum. The cells were first cultured in DMEM for 24 h before each experiment and then subjected to different treatment conditions.

### 4.4. Cell Viability

After seeding 2.5 × 10^4^ cells per well in 96-well plates, different concentrations of SWTI (0–1000 μg/mL) were added to the cell lines and they were incubated for 24 h. An MTT assay kit was used to evaluate the cell viability (Med-ChemExpress, HY-15924, NJ, USA) by incubating the cells for at least 3 h, followed by reading the absorbance at 570 nm using an ELISA plate reader (Molecular Devices, San Jose, CA, USA).

### 4.5. Western Blot Analysis

Proteins were extracted from the cells or tissues in GENESTAR (Kaohsiung, Taiwan) by homogenizing a RIPA buffer and then centrifuged at 10,000× *g* for 10 min at 4 °C. The Bio-Rad protein assay kit was employed for the quantification of the supernatant (BioRad, Hercules, CA, USA). Gel electrophoresis was used to separate protein samples containing equal amounts of protein (90 min at 100 V), followed by transfer onto a membrane at 200 mA for 2 h. After blocking, the membrane was incubated with primary antibodies against ACE2 (GTX101395) and TMPRSS2 (GTX100743), obtained from Genetex (San Antonio, TX, USA), followed by incubation with HRP-conjugated secondary antibodies (goat anti-rabbit IgG antibody). An ECL substrate was utilized to detect protein signals (Merck, Branchburg, NJ, USA). The Kodak Molecular Imaging Software Software 5.0 (East-man Kodak Company, Rochester, NY, USA) was utilized to calculate the protein expression.

### 4.6. Mouse Model

Female C57BL/6 mice were sourced from BioLASCO Taiwan Co., with weights ranging between 18 and 20 g and an age of 6–8 weeks. Three groups of mice (*n* = 6) were administered different treatments for 14 days via oral gavage: Group I received only distilled water as the control, Group II received 0.5 g/kg SWTI, and Group III received 1.0 g/kg SWTI. The mice were weighed at Day 0, Day 7, and Day 14 and were then sacrificed after 14 days, with tissues collected for further analysis. The Animal Management Committee of China Medical University’s regulations were followed during the animals’ care and the experiments (IACUC approval number: ǺCMUIACUC-2023-310).

### 4.7. Histopathological Analysis

The morphologies of the tissues and their damage were evaluated after fixation in formalin, embedding in paraffin, sectioning, and staining with H&E. The sections were examined and imaged using a microscope (Nikon, ECLIPSE, TS100, Tokyo, Japan).

### 4.8. Immunohistochemistry (IHC)

Immunohistochemical staining using ACE2 (bs-1004R, Bioss Inc., Woburn, MA, USA) or TMPRSS2 (ab214462, Abcam, Cambridge, MA) antibodies was carried out on tissue sections that were fixed in formalin and embedded in paraffin. The visual inspection and image capture of the sections were conducted using a microscope (Nikon, ECLIPSE, TS100, Japan). The ImageJ 1.54h software (NIH, USA) was utilized to quantify the stained areas.

### 4.9. Statistical Analyses

The statistical analysis was carried out with the assistance of the SPSS software version 21.0 (SPSS, Inc., Chicago, IL, USA), and the data were displayed as the mean ± standard deviation (S.D.). For the statistical analysis, an unpaired two-tailed Student’s *t*-test was conducted to establish the significance between the two groups, and a one-way analysis of variance (ANOVA) followed by the Scheffé test was used for comparisons among more than two groups. A *p*-value less than 0.05 was considered statistically significant.

## 5. Conclusions

As a result of the SARS-CoV-2 outbreak, consumers’ interest in healthy foods has surged, and their expectations regarding functional benefits such as body function regulation have significantly increased. The evidence provided by our study indicates that SBTI could be considered in a potential dietary intervention, given its ability to significantly reduce ACE2 and TMPRSS2 expression in both cellular and mouse models.

## Data Availability

Data are provided within the article.

## References

[1] Lefrançois T., Malvy D., Atlani-Duault L., Benamouzig D., Druais P.L., Yazdanpanah Y., Delfraissy J.F., Lina B. (2023). After 2 years of the COVID-19 pandemic, translating One Health into action is urgent. Lancet.

[2] Datta P.K., Liu F., Fischer T., Rappaport J., Qin X. (2020). SARS-CoV-2 pandemic and research gaps: Understanding SARS-CoV-2 interaction with the ACE2 receptor and implications for therapy. Theranostics.

[3] Pecoraro V., Cuccorese M., Trenti T. (2023). Genetic polymorphisms of ACE1, ACE2, IFTM3, TMPRSS2 and TNFα genes associated with susceptibility and severity of SARS-CoV-2 infection: A systematic review and meta-analysis. Clin. Exp. Med..

[4] Li L.Q., Huang T., Wang Y.Q., Wang Z.P., Liang Y., Huang T.B., Zhang H.Y., Sun W., Wang Y. (2020). COVID-19 patients’ clinical characteristics, discharge rate, and fatality rate of meta-analysis. J. Med. Virol..

[5] Yuan Y., Jiao B., Qu L., Yang D., Liu R. (2023). The development of COVID-19 treatment. Front. Immunol..

[6] Zamorano C.N., Grandvaux N. (2020). ACE2: Evidence of role as entry receptor for SARS-CoV-2 and implications in comorbidities. Elife.

[7] Dong M., Zhang J., Ma X., Tan J., Chen L., Liu S., Xin Y., Zhuang L. (2020). ACE2, TMPRSS2 distribution and extrapulmonary organ injury in patients with COVID-19. Biomed. Pharmacother..

[8] Hoffmann M., Kleine-Weber H., Schroeder S., Krüger N., Herrler T., Erichsen S., Schiergens T., Herrler G., Wu N., Nitsche A. (2020). SARS-CoV-2 cell entry depends on ACE2 and TMPRSS2 and is blocked by a clinically proven protease inhibitor. Cell.

[9] Hu B., Huang S., Yin L. (2021). The cytokine storm and COVID-19. J. Med. Virol..

[10] Manik M., Singh R.K. (2022). Role of toll-like receptors in modulation of cytokine storm signaling in SARS-CoV-2-induced COVID-19. J. Med. Virol..

[11] Banerjee R., Perera L., Tillekeratne L.M.V. (2021). Potential SARS-CoV-2 main protease inhibitors. Drug Discov. Today.

[12] Guerra Y., Celi D., Cueva P., Perez-Castillo Y., Giampieri F., Alvarez-Suarez J.M., Tejera E. (2022). Critical review of plant-derived compounds as possible inhibitors of SARS-CoV-2 proteases: A comparison with experimentally validated molecules. ACS Omega.

[13] Nguyen H.C., Chen C.C., Lin K.H., Chao P.Y., Lin H.H., Huang M.Y. (2021). Bioactive compounds, antioxidants, and health benefits of sweet potato leaves. Molecules.

[14] Huang G.J., Sheu M.J., Chen H.J., Chang Y.S., Lin Y.H. (2007). Growth inhibition and induction of apoptosis in NB4 promyelocytic leukemia cells by trypsin inhibitor from sweet potato storage roots. J. Agric. Food Chem..

[15] Jaw K.S., Chou L.H., Chang S.M., Duan K.J. (2007). Purification of a trypsin inhibitor from sweet potato in an aqueous two phase system. Biotechnol. Lett..

[16] Huang G.J., Ho Y.L., Chen H.J., Chang Y.S., Huang S.S., Hung H.J., Lin Y.H. (2008). Sweet potato storage root trypsin inhibitor and their peptic hydrolysates exhibited angiotensin converting enzyme inhibitory activity in vitro. Bot. Stud..

[17] Zhang J.J., Dong X., Liu G.H., Gao Y.D. (2023). Risk and Protective Factors for COVID-19 Morbidity, Severity, and Mortality. Clin. Rev. Allergy Immunol..

[18] Lin J.G., Huang G.J., Su Y.C. (2023). Efficacy analysis and research progress of complementary and alternative medicines in the adjuvant treatment of COVID-19. J. Biomed. Sci..

[19] Su Y.C., Huang G.J., Lin J.G. (2022). Chinese herbal prescriptions for COVID-19 management: Special reference to Taiwan Chingguan Yihau (NRICM101). Front. Pharmacol..

[20] Chien L.H., Deng J.S., Jiang W.P., Chen C.C., Chou Y.N., Lin J.G., Huang G.J. (2022). Study on the potential of *Sanghuangporus sanghuang* and its components as COVID-19 spike protein receptor binding domain inhibitors. Biomed. Pharmacother..

[21] Sun T.K., Huang W.C., Sun Y.W., Deng J.S., Chien L.H., Chou Y.N., Jiang W.P., Lin J.G., Huang G.J. (2022). Schizophyllum commune Reduces Expression of the SARS-CoV-2 Receptors ACE2 and TMPRSS2. Int. J. Mol. Sci..

[22] Wu C.-Y., Lin Y.-S., Yang Y.-H., Shu L.-H., Cheng Y.-C., Te Liu H. (2020). GB-2 inhibits ACE2 and TMPRSS2 expression: In vivo and in vitro studies. Biomed. Pharmacother..

[23] Ziegler C.G., Allon S.J., Nyquist S.K., Mbano I.M., Miao V.N., Tzouanas C.N., Cao Y., Yousif A.S., Bals J., Hauser B.M. (2020). SARS-CoV-2 receptor ACE2 is an interferon-stimulated gene in human airway epithelial cells and is detected in specific cell subsets across tissues. Cell.

[24] Bourgonje A.R., Abdulle A.E., Timens W., Hillebrands J.L., Navis G.J., Gordijn S.J., Bolling M.C., Dijkstra G., Voors A.A., Osterhaus A.D. (2020). Angiotensin-converting enzyme 2 (ACE2), SARS-CoV-2 and the pathophysiology of coronavirus disease 2019 (COVID-19). J. Pathol..

[25] Hikmet F., Méar L., Edvinsson Å., Micke P., Uhlén M., Lindskog C. (2020). The protein expression profile of ACE2 in human tissues. Mol. Syst. Bio.

[26] Sun Y.J., Velez G., Parsons D.E., Li K., Ortiz M.E., Sharma S., McCray P.B., Bassuk A.G., Mahajan V.B. (2021). Structure-based phylogeny identifies avoralstat as a TMPRSS2 inhibitor that prevents SARS-CoV-2 infection in mice. J. Clin. Investig..

[27] Razeghian-Jahromi I., Zibaeenezhad M.J., Lu Z., Zahra E., Mahboobeh R., Lionetti V. (2021). Angiotensin-converting enzyme 2: A double-edged sword in COVID-19 patients with an increased risk of heart failure. Heart Fail. Rev..

[28] Koch J., Uckeley Z.M., Doldan P., Stanifer M., Boulant S., Lozach P.Y. (2021). TMPRSS2 expression dictates the entry route used by SARS-CoV-2 to infect host cells. EMBO J..

[29] Ragia G., Manolopoulos V.G. (2020). Inhibition of SARS-CoV-2 entry through the ACE2/TMPRSS2 pathway: A promising approach for uncovering early COVID-19 drug therapies. Eur. J. Clin. Pharmacol..

[30] Li K., Meyerholz D.K., Bartlett J.A., McCray P.B. (2021). The TMPRSS2 inhibitor Nafamostat reduces SARS-CoV-2 pulmonary infection in mouse models of COVID-19. mBio.

[31] Files D.C., Gibbs K.W., Schaich C.L., Collins S.P., Gwathmey T.M., Casey J.D., Self W.H., Chappell M.C. (2021). A pilot study to assess the circulating renin-angiotensin system in COVID-19 acute respiratory failure. Am. J. Physiol. Lung Cell Mol. Physiol..

[32] Beyerstedt S., Casaro E.B., Rangel É.B. (2021). COVID-19: Angiotensin-converting enzyme 2 (ACE2) expression and tissue susceptibility to SARS-CoV-2 infection. Eur. J. Clin. Microbiol. Infect. Dis..

[33] Chen Y.R., Jiang W.P., Deng J.S., Chou Y.N., Wu Y.B., Liang H.J., Lin J.G., Huang G.J. (2023). *Anisomeles indica* extracts and their constituents suppress the protein expression of ACE2 and TMPRSS2 in vivo and in vitro. Int. J. Mol. Sci..

[34] Nayak S.S., Naidu A., Sudhakaran S.L., Vino S., Selvaraj G. (2023). Prospects of Novel and Repurposed Immunomodulatory Drugs against Acute Respiratory Distress Syndrome (ARDS) Associated with COVID-19 Disease. J. Pers. Med..

[35] Alessandri F., Di Nardo M., Ramanathan K., Brodie D., MacLaren G. (2023). Extracorporeal membrane oxygenation for COVID-19-related acute respiratory distress syndrome: A narrative review. J. Intensive Care..

[36] Yuan X., Pan C., Xie J., Qiu H., Liu L. (2022). An expanded definition of acute respiratory distress syndrome: Challenging the status quo. J. Intensive Med..

[37] Cid-Gallegos M.S., Corzo-Ríos L.J., Jiménez-Martínez C., Sánchez-Chino X.M. (2022). Protease inhibitors from plants as therapeutic agents—A review. Plant Foods Hum. Nutr..

[38] Hellinger R., Gruber C.W. (2019). Peptide-based protease inhibitors from plants. Drug Discov. Today.

[39] Shigetomi H., Onogi A., Kajiwara H., Yoshida S., Furukawa N., Haruta S., Tanase Y., Kanayama S., Noguchi T., Yamada Y. (2010). Anti-inflammatory actions of serine protease inhibitors containing the Kunitz domain. Inflamm. Res..

[40] Sabbah D.A., Hajjo R., Bardaweel S.K., Zhong H.A. (2021). An Updated Review on SARS-CoV-2 Main Proteinase (MPro): Protein Structure and Small-Molecule Inhibitors. Curr. Top. Med. Chem..

[41] Ahmad I., Pawara R., Surana S., Patel H. (2021). The repurposed ACE2 inhibitors: SARS-CoV-2 entry blockers of COVID-19. Top. Curr. Chem..

[42] Vangeel L., Chiu W., De Jonghe S., Maes P., Slechten B., Raymenants J., André E., Leyssen P., Neyts J., Jochmans D. (2022). Remdesivir, molnupiravir and nirmatrelvir remain active against SARS-CoV-2 Omicron and other variants of concern. Antivir. Res..

[43] Reina J., Iglesias C. (2022). Nirmatrelvir plus ritonavir (Paxlovid) a potent SARS-CoV-2 3CLpro protease inhibitor combination. Rev. Esp. Quimioter..

[44] Uzunova K., Filipova E., Pavlova V., Vekov T. (2020). Insights into antiviral mechanisms of remdesivir, lopinavir/ritonavir and chloroquine/hydroxychloroquine affecting the new SARS-CoV-2. Biomed. Pharmacother..

[45] Erlanger B.F., Kokowsky N., Cohen W. (1961). The preparation and properties of two new chromogenic substrates of trypsin. Arch. Biochem. Biophys..

